# The Southpaw Advantage? - Lateral Preference in Mixed Martial Arts

**DOI:** 10.1371/journal.pone.0079793

**Published:** 2013-11-19

**Authors:** Joseph Baker, Jörg Schorer

**Affiliations:** 1 School of Kinesiology and Health Science, York University, Toronto, Canada; 2 Institute of Sport Science, University of Oldenburg, Oldenburg, Germany; VU University Amsterdam, The Netherlands

## Abstract

Performers with a left-orientation have a greater likelihood of obtaining elite levels of performance in many interactive sports. This study examined whether combat stance orientation was related to skill and success in Mixed Martial Arts fighters. Data were extracted for 1468 mixed martial artists from a reliable and valid online data source. Measures included fighting stance, win percentage and an ordinal measure of skill based on number of fights. The overall analysis revealed that the fraction of fighters using a southpaw stance was greater than the fraction of left-handers in the general population, but the relationship between stance and hand-preference is not well-understood. Furthermore, t-tests found no statistically significant relationship between laterality and winning percentage, although there was a significant difference between stances for number of fights. Southpaw fighters had a greater number of fights than those using an orthodox stance. These results contribute to an expanding database on the influence of laterality on sport performance and a relatively limited database on variables associated with success in mixed martial arts.

## Introduction

Laterality refers to a preference for one side of the body over the other, most commonly reflected in right- or left-handedness. Researchers have shown that the proportion of ‘lefties’ in the general population has remained stable over 10,000 years [1] and the stability of this effect over time suggests some consistent advantage to being left-handed, otherwise evolutionary mechanisms would have removed this polymorphism from the population. Sport may reflect an environment where these advantages are demonstrated; for instance, left-handedness is associated with a greater likelihood of obtaining elite levels of performance in many interactive sports including baseball [2] and tennis [3] with significant over-representations of left-handed players at the highest levels of competition. The negative perceptual frequency hypothesis proposes that these players have an advantage in sports where athletes must rapidly respond to dynamic environments, such as tennis and fencing, because the decision-making heuristics athletes use to anticipate their opponent’s actions are based on an extensive duration of training and competition against right-handers with comparatively less exposure to left-handers [4]–[6]. Support for this proposed mechanism comes from reviews showing that laterality effects are restricted to sports where performers are required to interact with opponents (e.g., tennis and ice hockey) and not in non-interactive sports (e.g., swimming, gymnastics and archery [1]).

Advantages of a left-sided orientation have also been found in combat sports like boxing [7], fencing [8] and judo [9]–[10], presumably because of the association between laterality and evolutionary connections between fighting ability and survival [7]. In this study, we consider whether laterality as represented by fighting stance varied with skill level and influenced performance outcomes in mixed martial arts (MMA), a combat sport that integrates grappling sports like wrestling, jui-jitsu and judo with striking sports like boxing, karate, taekwondo, and kick-boxing. Based on prior work in boxing [11], we hypothesized that a left-orientation, that is, a ‘southpaw’ stance where fighters lead with their right hand, would be associated with skill level. This would be reflected in a greater proportion of southpaws among fighters with more fights (a measure of skill level in MMA). Additionally, given the greater success of left-oriented performers in many sports, we hypothesized that left-oriented fighters would have higher winning percentages than right-oriented fighters.

## Materials and Methods

Data for 2053 MMA fighters were obtained from a valid and reliable online data source (fightmetric.com, see website for more information regarding validity and reliability). Of these, 1468 had data for fighting stance and were retained for further analysis. Even though this study used data freely available in the public domain, all ethical recommendations regarding confidentiality and anonymity were followed when collecting, analyzing and reporting study results [12]. Variables included combat stance, wins, losses and draws. These latter variables were used to compute win percentage for each athlete. Combat stance included four orientations including orthodox, southpaw, switch, and open; however, due to low numbers in the latter two categories (totaling just over two percent), statistical analyses were limited to comparisons between orthodox and southpaw groups. In MMA, skill level is somewhat difficult to determine. Initially, we considered simply using ‘winning percentage’; however, this variable is confounded by skill level of the opponent and number of fights. Moreover, fighters who lost close decisions are often kept within the system while those who have been soundly defeated are usually removed from further opportunities for competition. As a result, we considered both winning percentage and number of fights in this sample. To ensure we did not confound our data by using winning percentage without accounting for number of fights (e.g., a fighter with 1 fight and 1 win would have a winning percentage of 100%), we compared winning percentage across groups of fighters categorized by number of fights (cf. Table 1). We created an ordinal variable that grouped number of fights into intervals of ten (e.g., 1–10, 11–20, and so on up to 70+ fights). Stance differences in number of fights and winning percentage were considered in the overall sample using independent samples t-tests. Additionally, we explored differences in winning percentages in the number of fight categories. Levenés test for equality of variances was administered and if necessary, adjustment values for the t-tests were used. Alpha was adjusted using Bonferroni’s correction (alpha<.025) and SPSS 21.0 was used for all statistical analyses. Effect sizes and test powers were calculated using G*power 3.1.6.

**Table 1:** Comparison of combat stances and winning percentage by number of fights.

Number of fights	Southpaw N (%)	Orthodox N (%)	Winning percentagesouthpaws (SD)	Winning percentageorthodox (SD)
Overall	256 (17.8)	1179 (82.2)	64.0 (20.4)	62.6 (21.3)
1–10	61 (16.9)	301 (83.1)	48.9 (29.8)	49.0 (31.7)
11–20	79 (15.7)	425 (84.3)	70.7 (14.4)	68.9 (13.9)
21–30	60 (19.0)	256 (81.0)	68.9 (12.6)	66.8 (12.5)
31–40	28 (19.9)	113 (80.1)	66.6 (14.2)	64.6 (13.0)
41–50	15 (21.7)	54 (78.3)	64.1 (9.6)	62.8 (12.6)
51–60	6 (25.0)	18 (75.0)	68.2 (12.0)	62.9 (9.6)
61–70	3 (33.3)	6 (66.7)	61.7 (17.1)	75.9 (4.8)
71<	4 (40.0)	6 (60.0)	62.6 (11.7)	61.2 (17.0)

## Results

The vast majority (80.3%) of MMA fighters reported using an orthodox stance with 17.4% reporting a southpaw stance (Table 1). Only 2.3% reported stances other than orthodox and southpaw. There were significant differences between stances in the number of fights, *t*(342.20) = 2.08, *p* = .02, *d* = .15. As predicted, the southpaw stance athletes had more fights, *M* = 22.01, *SD* = 16.52, than orthodox fighters, *M* = 19.69, *SD* = 14.26. The t-test examining winning percentages for orthodox versus southpaw stances indicated no differences, *t*(1433) = 0.97, *p* = .33, d = .06, 1–β = .17, although the winning percentage of the southpaws, *M* = 64.0, *SD* = 20.4, was slightly higher than for orthodox fighters, *M* = 62.6, *SD* = 21.3.

## Discussion

This study extends our understanding of the role of lateral preference and performance in interactive sports. There was an increased proportion of southpaw (i.e., left-oriented) fighters overall compared to proportions of left-handed individuals in the general population (i.e., 17% southpaws versus between 10–12% left-handers in the general population as reported by Raymond et al. [7]); however, the relationship between fighting stance and handedness is not well understood. For example, Oscar De La Hoya, arguably the most successful boxer in history, is left-hand dominant but boxes from an orthodox stance. Therefore, it was not possible to determine whether this is an over-representation of southpaw stances similar to that found for handedness in other studies on laterality [3], [5], [8]. As previously noted in ice hockey [12], normative data for sport-based laterality tasks (e.g., shooting side in ice hockey, combat stance in martial arts and boxing, hitting side in baseball) would be valuable for determining the strength of the relationship between handedness and other lateral preferences. Future studies are necessary to compare the proportions of athletes utilizing different stances at lower levels of skill to add further depth to our understanding of fighting stance in MMA.

Interestingly, our analysis suggests that skill in MMA is more nuanced than other combat sports. When we considered winning percentage, there were no clear differences between southpaw and orthodox stances overall or when considered relative to number of fights. However, when skill level was operationalized as number of fights, there was a significant difference between stances. Although the effect size was small, it indicated that as number of fights increased the proportion of southpaw stances also increased.

This study highlighted several avenues for future research. A factor not controlled in the current analyses was the complex interaction between striking and grappling in determining success in MMA compared to other combat sports where these factors operate in isolation (e.g., grappling in judo or striking in boxing). In addition, research examining laterality differences across the different methods of attaining success (e.g., submission, technical knockout, or judges scorecard) may provide additional insight. It is also not clear from the present analysis whether ‘success’ in MMA (i.e., an increasing number of MMA fights) is ultimately the result of a greater number of lateralized attacks or defensive responses (i.e., initiation versus reaction). This highlights a limitation of using lateral preference to understand skilled performance in complex environments such as MMA. Further work examining the precise perceptual-cognitive skills and strategies that underpin the ‘southpaw advantage’ would be valuable for understanding this effect (for an example of this work in other interactive sports see [6], [14]). Furthermore, given the relative ‘immaturity’ of MMA as a sport and martial art, an intriguing question is whether this laterality effect will persist over time or whether MMA, like other ‘evolving systems’ will stabilize over time where variables important for influencing performance early in a sport’s development meet a level of optimal balance and are no longer predictive to the same extent (see [13]). This argument was recently made by Loffing et al. [14] to explain the lack of laterality differences in contemporary tennis, a sport that has shown laterality effect in the past.

## Conclusions

Collectively, these results contribute to an expanding database on the influence of laterality on sport performance and a relatively limited database on predictive variables in MMA performers. Further research should continue the examination of laterality in the component sports of MMA to better understand the effects found in the current study. Moreover, investigations describing the specific training behaviors used by MMA athletes may provide useful data regarding the relationship between combat stance and performance in combat sports as well as informing training and competition strategies to reduce the ‘southpaw advantage’.
